# Application of Eye Tracking Technology in Aviation, Maritime, and Construction Industries: A Systematic Review

**DOI:** 10.3390/s21134289

**Published:** 2021-06-23

**Authors:** Daniel Martinez-Marquez, Sravan Pingali, Kriengsak Panuwatwanich, Rodney A. Stewart, Sherif Mohamed

**Affiliations:** 1 School of Engineering and Built Environment, Griffith University, Gold Coast, QLD 4222, Australia; s.pingali@griffith.edu.au (S.P.); r.stewart@griffith.edu.au (R.A.S.); s.mohamed@griffith.edu.au (S.M.); 2 School of Civil Engineering and Technology, Sirindhorn International Institute of Technology, Thammasat University, Pathum Thani 12120, Thailand; kriengsak@siit.tu.ac.th

**Keywords:** eye tracking, visual attention, human-machine interfaces, mental performance, accidents, hazard identification, training, construction, aviation, maritime

## Abstract

Most accidents in the aviation, maritime, and construction industries are caused by human error, which can be traced back to impaired mental performance and attention failure. In 1596, Du Laurens, a French anatomist and medical scientist, said that the eyes are the windows of the mind. Eye tracking research dates back almost 150 years and it has been widely used in different fields for several purposes. Overall, eye tracking technologies provide the means to capture in real time a variety of eye movements that reflect different human cognitive, emotional, and physiological states, which can be used to gain a wider understanding of the human mind in different scenarios. This systematic literature review explored the different applications of eye tracking research in three high-risk industries, namely aviation, maritime, and construction. The results of this research uncovered the demographic distribution and applications of eye tracking research, as well as the different technologies that have been integrated to study the visual, cognitive, and attentional aspects of human mental performance. Moreover, different research gaps and potential future research directions were highlighted in relation to the usage of additional technologies to support, validate, and enhance eye tracking research to better understand human mental performance.

## 1. Introduction

### 1.1. Eye Tracking in High-Risk Industries

Industries with work environments and processes that pose a considerable risk of harm to people and nature are defined as high-risk industries [1]. Some of these high-risk industries include aviation, maritime, and construction [2,3]. Most accidents in the aviation, maritime, and construction industries are caused by human error [4,5,6,7,8]. Indeed, the National Transportation Safety Board survey found that 88% of aviation accidents between 1989 and 1992 were caused by human error [7]. According to the International Maritime Organisation (IMO), human error is also the major cause of incidents in the maritime industry, accounting for 85% of all industry accidents [8]. The construction industry is one of the most hazardous industries globally [9]. For example, though it comprises only 5% of the work force in the US, the construction industry accounted for almost 20% of workplace deaths among all industries between 2003 and 2012. Similar trends are shown in Australia and the UK [10]. These three industries are some of the most critical sectors in the global economy. Furthermore, they are exposed to numerous occupational risks that are very challenging to control and mitigate. These risks entail large inherent losses but also massive profits [11].

Most accidents in the aviation, maritime, and construction industries can be traced back to impaired mental performance as a result of attention failure [5]. Human mental performance can be affected by a variety of external and internal factors [5], such as emotional state, risk perception, training, and human-machine interfaces (HMIs). Almost half of the brain’s neural pathways are used for visual processing [12], making vision a key element in mental performance [13]. As such, an effective method for understanding the factors that affect human performance is the analysis of visual information processing [14]. Several psychology and neuroscience studies have concluded that eye movement can help in understanding the visual, cognitive, and attentional aspects of human performance [15]. A good tool for measuring eye movement and eye position information is by using an eye tracking device. This unique tool allows eye movement information to be recorded, which can help to assess an individual’s mental state, to understand cognitive processing and behaviour, and to interpret individuals’ responses to different visual stimuli [16]. 

Eye tracking research dates back almost 150 years [17] and has been widely used in different fields for numerous purposes [18,19]. Eye tracking technologies have been used in different industries for the evaluation of present and future working environments, especially when they involve high degrees of risk. For example, eye tracking has been used in medicine and surgery as well as the aviation and maritime industries to improve teaching strategies and trainee performance [20,21,22]. To enhance safety, eye tracking has also been used to understand construction workers’ risk perception and hazard identification [23]. 

Eye tracking technologies can also be integrated with various other technologies, such as simulators, motion capture devices, and augmented reality, to better understand individuals’ gaze patterns during different scenarios. For example, in the automobile, aviation, and maritime industries, eye tracking technologies have been used in conjunction with sophisticated simulators to understand where pilots and navigators are looking under different physical and mental states, such as fatigue, anxiety, and stress [24,25]. Furthermore, the impacts of HMIs such as cockpits (for airplanes) and bridges (for ships) on human visual attention and their performance have been studied using eye trackers [26,27].

### 1.2. Purpose and Objectives

Overall, eye tracking technologies provide the means to capture a variety of eye movement information that reflects different human cognitive, emotional, and physiological states in real time, which can be used to gain a wider understanding of the human mind in different scenarios. The purpose of this systematic literature review is to explore how eye tracking technologies have been used in high-risk industries such as the aviation, maritime, and construction industries and to uncover current eye tracking research gaps in these industries. Thus, the objectives of this study are as follows:

- To perform a demographic analysis to identify the main countries that are using eye tracking research with applications in aviation, maritime, and construction fields;
- To identify the main applications of eye tracking research in the aviation, maritime, and construction industries;
- To identify the different human aspects that are studied in eye tracking research in aviation, maritime and construction scenarios;
- To identify the technologies that are integrated with eye tracking devices to study the different human aspects in aviation, maritime, and construction scenarios; and
- To determine research gaps in the development and application of eye tracking technologies within the aviation, maritime, and construction industries.

## 2. Materials and Methods

A systematic search was conducted on 17 August 2020 using the Science Direct and Google Scholar databases following the preferred reporting items for systematic reviews and meta-analyses (PRISMA) statement [28]. In this systematic literature search, the keywords ‘eye move*’, ‘eye track*’, ‘gaze move*’, and ‘gaze track*’ were connected with the Boolean operator ‘“OR”’ and accompanied by the following keywords using the ‘“AND”’ Boolean operator: ‘maritime’, ‘aviation’, ‘construction’.

Three full phrases were used:

(‘eye move*’ OR ‘eye track*’ OR ‘gaze move*’ OR ‘gaze track*’) AND maritime;

(‘eye move*’ OR ‘eye track*’ OR ‘gaze move*’ OR ‘gaze track*’) AND aviation;

(‘eye move*’ OR ‘eye track*’ OR ‘gaze move*’ OR ‘gaze track*’) AND construction.

### 2.1. Selection Criteria

**Figure 1:**
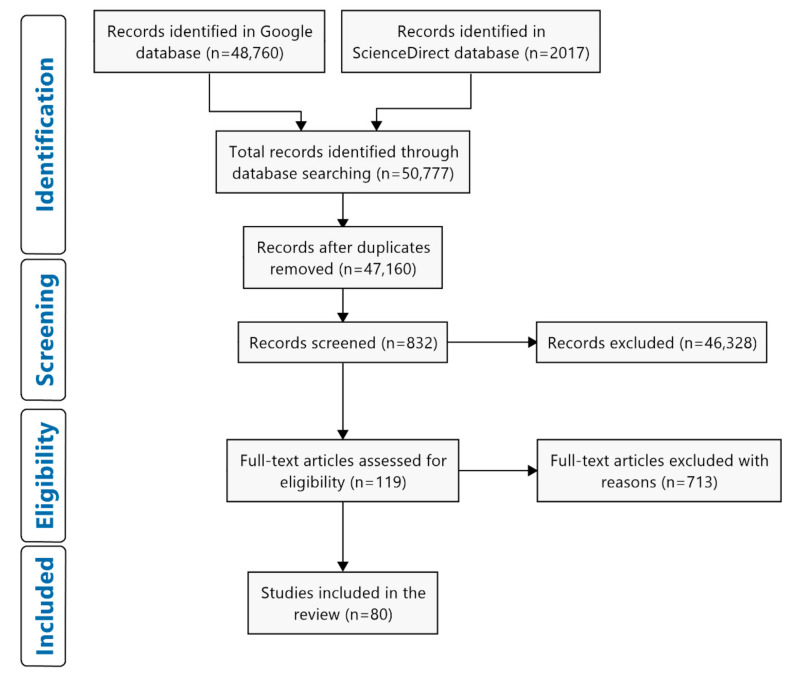
Search strategy and study selection.

The initial literature search returned a total of 50,777 articles, from which a total of 3617 duplicated articles were removed, as presented in Table 1. We collected the first 10 pages of search results (sorted by relevance) for each search phrase, yielding a total of 832 articles. These 832 articles were screened according to the following inclusion criteria: (1) conference and peer-reviewed papers with the full text published within the last 20 years (2000 to 2020), (2) empirical studies reporting the use of eye tracking technologies, and (3) published in the English language. This resulted in 119 articles eligible for full-text assessment. In the full-text checking process, review articles, as well as articles that were not related to the aviation, construction, and maritime industries, were excluded. The full-text checking process determined that 80 articles met the inclusion criteria for this review, as shown in Figure 1.

### 2.2. Data Extraction and Analysis

The systematic literature search aimed to identify the different applications and methodologies used in eye tracking research in three high-risk industries. The classification topics were as follows: maritime, aviation, construction, year of publication, location of publication, industry application, type of scenario (e.g., real environment or simulation), cognitive (Cog), emotional (Emo), physiological (Phys), integration with other technologies, and types of eye tracking devices. Full-text screening was performed by D.M. and S.P. to avoid potential bias. A consensus meeting resolved any discrepancies between reviewers. Once all applicable literature was identified, the extracted data were analysed by normalising the number of studies according to the classification topics previously described.

## 3. Results

The systematic literature search identified a total of 80 studies that fulfilled the inclusion criteria. An initial data classification was performed as described previously (see Table 2). After this, a more detailed analysis was performed in which a total of 2240 qualitative data items were extracted and synthesised to uncover industry distribution and demographics as well as the different applications of eye tracking studies in the maritime, aviation, and construction industries.

This section first defines eye tracking studies regarding the aviation, maritime, and construction industries with respect to industry distribution and demographics. These studies were synthesised to uncover the countries that apply eye tracking in the three industries. Moreover, we also sought to uncover and explain how eye tracking technologies are used in these three industries. Particular attention has been devoted to the following aspects: the different applications of eye tracking studies; the different cognitive, emotional, and physiological processes studied in eye tracking research; the preferred types of eye tracking devices used; and the different technologies that are integrated to enhance the capabilities of eye tracking research.

### 3.1. Demographics

**Figure 2:**
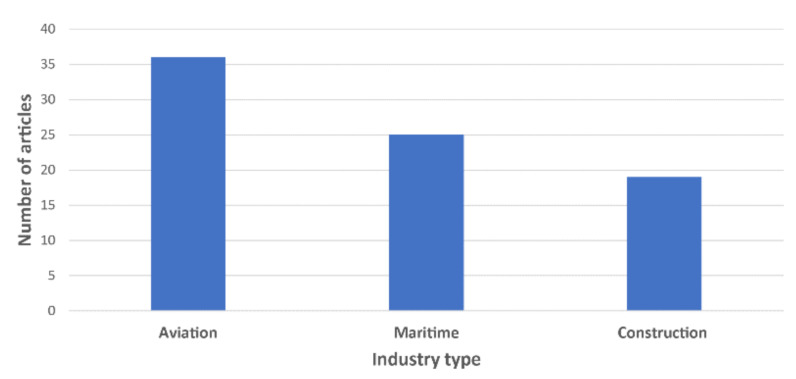
The distribution of eye tracking research articles by industry type.

**Figure 3:**
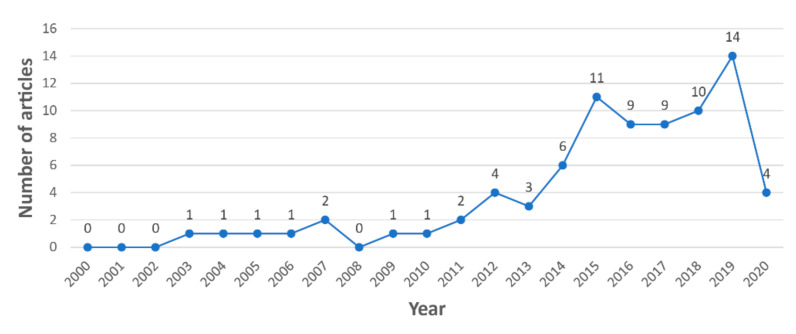
Temporal trends of eye tracking research articles in the aviation, maritime, and construction industries published between 2000 and 2020 (N = 80).

**Figure 4:**
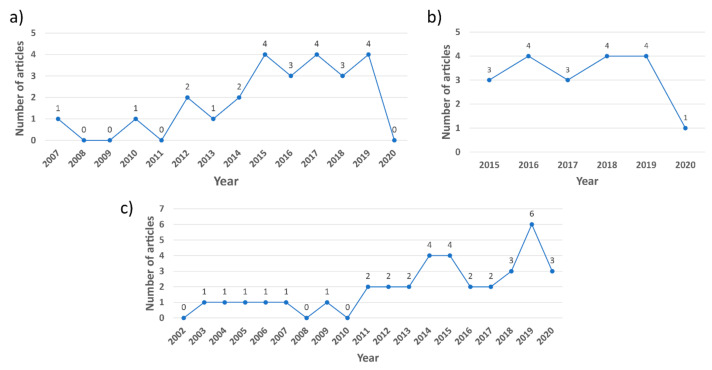
Temporal trends of the number of eye tracking research articles per year in the (**a**) maritime industry (N = 25), (**b**) construction industry (N = 19), and (**c**) aviation industry (N = 36).

According to Figure 2, the highest number of studies identified in the systematic literature search relate to the aviation industry (36 studies), followed by the maritime and construction industries (25 and 19 studies, respectively). In general, the use of eye tracking technologies in these three industries increased from 2003 to 2020, as shown in Figure 3. Almost 70% of the 80 reviewed articles were published in the last five years (Figure 3). According to Figure 4a,c, research interest in eye tracking in the maritime and aviation industries dates back more than 13 years. In contrast, the application of eye tracking technologies only recently started to attract interest in the construction industry (Figure 4b), where the oldest article identified was published in 2015. This limited interest is also reflected by the low number of eye tracking studies in the construction industry identified in this study.

In terms of demographic distribution, researchers conducting eye tracking studies in the three high-risk industries were located in Europe (55%), North America (28%), Asia (14%), Australia (1%), the Middle East (1%), and South America (1%; Table 3). Although the studies were distributed among different locations, overall, the USA was the leader in eye tracking studies (20 articles), followed by Germany and Norway (both with 10 articles), and then China and the UK (both with seven articles).

**Figure 5:**
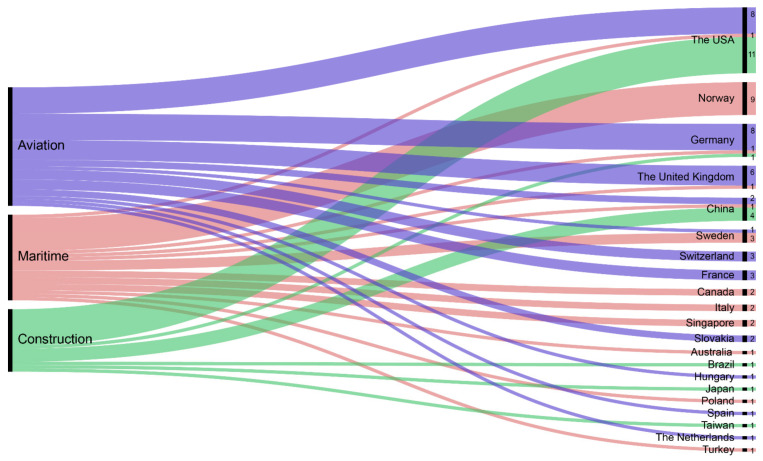
Distribution of identified studies according to industry and number of articles per region (created with RAWGraphs [104]).

Regarding the distribution of eye tracking studies per location in the aviation industry, the USA and Germany were in first place, followed by the UK (see Figure 5). These results are unsurprising since Boeing, the world’s foremost aircraft manufacturer from 2012 to 2020, is an American company [96]. Germany holds the major production sites of Airbus, which Boeing surpassed in 2020 to become the largest aircraft manufacturer company in the world [97,98]. The UK has the second largest aircraft manufacturing industry worldwide and is known as a global centre of excellence for the design and production of turbines, helicopters, and aircraft components and systems [99].

In the maritime industry, eye tracking research is dominated by Norway (nine articles), followed by Sweden (three articles) and Canada, Italy, and Singapore (two articles each). These results correlate with the importance of the maritime sector in these countries. For example, Norway’s economy heavily relies on its strong maritime activities, and the region currently has several leading shipping companies and the world’s largest shipping stock exchange [11,100]. However, despite the substantial dependence of Canada, Italy, and Singapore on marine transportation, there is a low number of eye tracking research studies in maritime applications. This trend can also be observed in Australia, Japan, and Taiwan, which are regions surrounded by water that nonetheless have one or no eye tracking studies in the maritime field. 

Regarding the construction industry, the USA is also the leading region in eye tracking research (11 articles), followed by China (four articles). It is important to note that the USA is one of the largest construction markets worldwide [101], and China’s urbanisation infrastructure has been experiencing exceptional growth in recent decades [102,103]. The USA, Germany, and China are the only regions that have published eye tracking studies in the aviation, maritime, and construction industries.

### 3.2. Eye Tracking Metrics

The visual system is considered to be the most important system of the human body after the brain and the foremost in terms of sensory systems, as 85% of the information that the organism obtains from the environment is processed through it [105]. In humans, the eyes play a very important role in communication. For example, eye contact and gaze direction are used to establish socio-emotional connection, indicate the target of our visual interest, or to regulate interaction [106]. Moreover, different cognitive processes and intentions can be reflected by our gaze behaviour (e.g., we often look at things before acting on them) [107]. In eye tracking research, different eye metrics have been identified and related to different cognitive, emotional, and physiological states, which can be used to gain a wider understanding of the human mind. These eye metrics are fixation, saccadic movements, pupillary response, and eye blink rate.

#### 3.2.1. Fixation

Fixation is generally associated with visual processing and information acquisition [108]. Fixation occurs when the eye remains still and the pupil is stationary for approximately 180–300 ms [33,62,108]. During a fixation, people obtain new information from an object, stimulus, or location [31]. Statistical analysis of different fixation metrics such as fixation frequency, fixation duration, fixation duration max, and standard deviation of the fixation duration are related to human performance as well as various cognitive attentional processes [34]. For example, a pilot’s situation awareness (SA) performance and expertise level can be inferred from the distribution of fixations and fixation duration on relevant areas of interest (AOIs) [26]. In aviation research, a study found that experienced pilots spend more time fixating on multiple flight instruments, without dwelling for too long on any single one [109]. However, experts of different backgrounds can have different gaze behaviours. For example, in the maritime industry, expert operators spend more time fixating on the outside environment, whereas novices focus more on the deck [34]. Independent of experience, this difference can also be observed in the aviation industry. Commercial pilots spend more time looking at the instrument panel, whereas military pilots spend more time looking through the window [109]. During a visual search activity, fixation duration can also be used to predict a subject’s hazard recognition [93]. In the construction industry, a study found that workers with a higher perception of risk fixated for a longer time on objects that pose a hazard when they are identified for first time [23]. Similarly, it was found that air traffic controllers with a higher fixation count on relevant AOIs have a higher failure detection frequency [59]. On the contrary, a short fixation duration is generally associated with individuals experiencing anxiety and a threat state [68].

#### 3.2.2. Saccades

Saccades are rapid eye movements that occur when a person shifts between fixations [43]. Saccades last around 10–100 ms, during which time visual information transfer is suppressed [110]; therefore, saccades are not directly related to cognitive processing [33]. However, saccade velocity can be related to lethargy, stress, and fatigue [64,93]. For example, saccade rate decreases with fatigue and difficult tasks [27]. Saccadic length has also been used to measure mental workload [33] and has been shown to increase with increased mental workload (MWL) [73], with very short saccades related to the presence of conflict [56]. In a maritime setting, the number of saccades can reveal improvements in the scanning technique of a navigator [111].

#### 3.2.3. Pupil Size

One of the most distinguishing features of the human eye is the pupil [112] and extracting information about the size and location of its centre is relatively easy using video recording [113]. Pupil size is influenced by illumination and regulates the amount of light that enters the retina. Pupil size is also affected by emotions, muscular fatigue, cognitive processes, and MWL [26]. For example, an increased pupillary dilation response has been related to increased processing load in different maritime settings [34,45]. Moreover, pupil size has been used to understand the cognitive effects of stress in aviation emergencies and conflict [56,64]. In quay crane operators, pupil dilation was used to measure alertness and fatigue evolution [24]. Visual fatigue caused by aircraft instrument displays has been studied by analysing the effects of electronic displays on pupil diameter [78]. In a simulated maritime environment, pupil dilation was shown to reveal the upcoming judgment of the human operator [38].

#### 3.2.4. Blink Rate

Blink rate refers to the number of blinks per second or minute and is related to physiological factors such as mood state, task demands, attention, and tension [34]. Blink rate is generally used to measure MWL and fatigue [48]. For example, high blink rates are correlated with fatigue and low MWL [31,113]. Regarding scanpaths, this eye tracking metric results from the combination of fixations and saccades. According to Bhoir et al. [85], ‘an optimal scanpath is a straight line eye movement to desired targets and a short fixation on targets’. A scanpath can provide information about a subject’s cognitive process for encoding information [70,114]. For example, a study found that with an increasing workload, subjects tend to use a less random scanpath [34]. In the aviation and maritime industries, subjects’ scanpath is used to understand how they interact and absorb information from their environment, such as computer interfaces and instrument panels [70]. A summary of the described eye metric characteristics can be found in Table 4.

### 3.3. Eye Tracking Data Visualisation Tools

**Figure 6:**
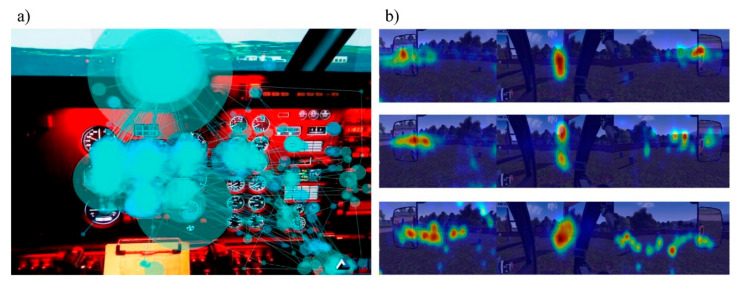
(**a**) Pilot scanpath of cockpit flight instruments during a landing approach [75]; (**b**) Excavator operator gaze point distribution heatmap [92].

Due to the large amount of information provided by eye tracking devices, eye tracking visualisation techniques provide additional insight when paired with comprehensive statistical analysis [50]. Visualisation techniques commonly used for representing eye tracking data are heatmaps and scan paths, as presented in Figure 6 [115]. 

Heatmaps are the main data visualisation tool used to analyse the accumulated positions of gaze and fixation distributions among AOIs [58,116]. A heatmap is a 2D visualisation of the analysed eye tracking data represented in colour scales [117]. In a heatmap, hot zones correspond to higher gaze and fixation densities, whereas cool zones correspond to lower densities [118]. Heatmaps can be used with data from an individual or from a group of people [89]. The limitation of heatmaps is that they only show density-based data and lack information about the sequential order of eye movements [117]. Another type of eye tracking visualisation tool is a scanpath. Scanpaths are used to compile the eye movements and are defined as a spatial arrangement of a sequence of saccade–fixation–saccade. Scanpaths are used to reveal the sequential order of observed areas [119] and provide information related to individuals’ search efficiency [120].

### 3.4. Overview of Types of Eye Tracking Methods

Currently, there are four main methods used to measure eye movements. These methods are Electro-OculoGraphy (EOG), scleral contact lens/search coil, Photo-OculoGraphy (POG) or Video-OculoGraphy (VOG), and video-based combined pupil–corneal reflection [49]. Electro-OculoGraphy (EOG) was the most used eye tracking method 50 years ago. The EOG method uses a series of electrodes placed around the eyes to measure the electric field in the tissue surrounding the eye. This electric field is caused by the electric potential difference between the cornea (transparent front part of the eye) and the ocular fundus (the interior surface of the eye) [121]. One of the main advantages of EOG is that light conditions have no impact on the quality of the eye recording. Moreover, the signal processing of EOG does not require any complex video and image processing [122]. 

Scleral contact lens/search coil is one of the most accurate eye movement measurement methods and is considered the gold standard in oculomotor research [123]. Scleral contact lens/search coil consists of a pair of contact lenses mounted with reflecting phosphors, line diagrams, or wire coils [121]. The working principle involves a coil of metallic wire that can be tracked when it moves through an electro-magnetic field [124]. The limitations of the scleral contact lens/search coil method include eye irritation and potential cornea damage. Therefore, this method has a limited experimental time of use of around 30 min per session [123]. Photo-oculography (POG) or video-oculography (VOG) comprise several eye movement tracking techniques and are based on the measurement of the relative position of the reflected image of an infrared source on the cornea and the pupil centre [49]. The disadvantage of POG or VOG is that the quality of measurements can be affected by head movements and blinking artifacts. Moreover, the measurement of ocular features provided by POG or VOG techniques can be extremely tedious and prone to error [125].

Although all the mentioned eye tracking techniques are, in general, suitable for eye movement measurements, they do not often provide point of regard measurement. To counteract this disadvantage, video-based trackers are equipped with high-resolution cameras and image processing hardware to measure the pupil centre and corneal reflection (of a light source, usually infrared). The combined pupil–corneal reflection method allows computation of the point of regard in real time [126]. The pupil is easily detected using the bright pupil phenomenon occurring when the eye is exposed to near-infrared light [127]. Advanced image processing algorithms and a physiological 3D model of the eye are then used to estimate the position of the eye in space and the point of gaze with high accuracy [128].

### 3.5. Types of Modern Video-Based Eye Tracking Devices

**Figure 7:**
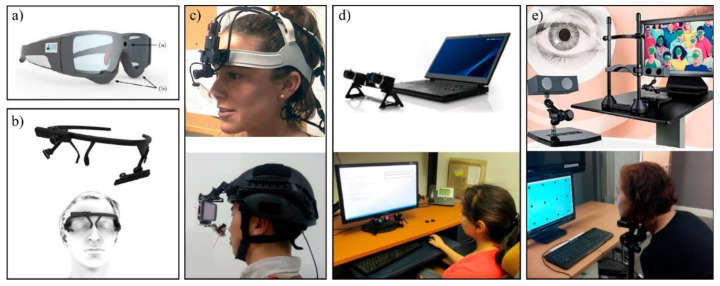
Example of the different types of eye tracking devices: (**a**) eye tracking glasses [75]; (**b**) headband [92]; (**c**) helmet-mounted [130,131]; (**d**); remote or table [132]; (**e**) tower-mounted [133,134].

Today, most eye tracking systems are video-based, with infrared illumination and an eye video camera [108]. Depending on the type of activity to be studied, the experiment, and the environment, different video-based eye tracking systems are required. Eye tracking systems can be categorised as remote, mobile, or tower-mounted based on how they interface with the user and environment, as presented in Figure 7 [129].

#### 3.5.1. Mobile Eye Tracking Devices

Mobile eye tracking devices are also referred to as head-mounted or wearable devices. Such devices usually have an additional scene camera that records the scene or field of view [135]. Mobile eye tracking devices are worn by the participant in the form of a headband, glasses, or a helmet-mounted system, allowing the participant to move freely in the experimental environment, as shown in Figure 7a–c [136]. Mobile devices are mainly binocular and are usually more accurate than remote devices [135]. On mobile devices, gaze tracking is performed relative to the entire field of view, which makes it ideal for real-world experiments [137]. Head- or helmet-mounted devices are less invasive and more comfortable, and they can be worn with other technologies such as electroencephalography (EEG) [138]. Nonetheless, mobile tracking devices have several limitations, such as difficulty in tracking eye movements in sunlight and being inappropriate for environments with heavy winds and water spray [32]. Moreover, eye movements to the periphery can be difficult to track and will often show less accuracy. Mobile tracking devices do not have an absolute coordinate system, instead requiring gaze data to be recorded in a coordinate system defined by the scene camera [138]. Finally, data inaccuracy can be introduced if the mobile eye tracking device does not properly fit the individual’s face [136].

#### 3.5.2. Remote Eye Tracking Systems

Remote eye tracking systems do not touch the person at all and measure the eye from a distance, as presented in Figure 7d [121]. This type of eye tracking device is mainly used for screen-based interaction experiments. The advantage of remote eye tracking systems is that the participant can use a computer completely naturally while the eye tracking system records data [137]. Moreover, data processing can be less complex and significantly more efficient compared with wearable systems because the visual space is already integrated [136]. Furthermore, remote eye tracking devices work very well with other research technologies because they do not touch the participant [138]. However, the limitations of remote eye tracking devices are that they can only be used with fixed working areas; this can result in gaps in data accuracy and artefacts when the participant excessively moves his/her head [138]; they are also intolerant to infrared (IR) sources such as sunlight, especially if the sun is reflected in the participant’s eyes. 

To further improve the accuracy of remote eye tracking systems, head-supporting towers are usually employed, as shown in Figure 7e. Head-supporting towers are in close contact with the participant via a bite bar or chin rest and therefore restrain head movements [137]. These devices allow the capture of the highest-quality data by restricting the participant’s head movement, although they are less realistic and natural. The saccade resolution of a remote eye tracker fitted with a head-supporting tower is two to five times greater than that of a remote/head-free eye tracker [16]. However, the restrictive setting of head-supporting towers limits their use for dynamic environments. As a result, they are mainly used in studies that require high precision and where the subject is usually looking at a fixed screen [139].

#### 3.5.3. Eye Tracker Performance and Data Quality

**Figure 8:**
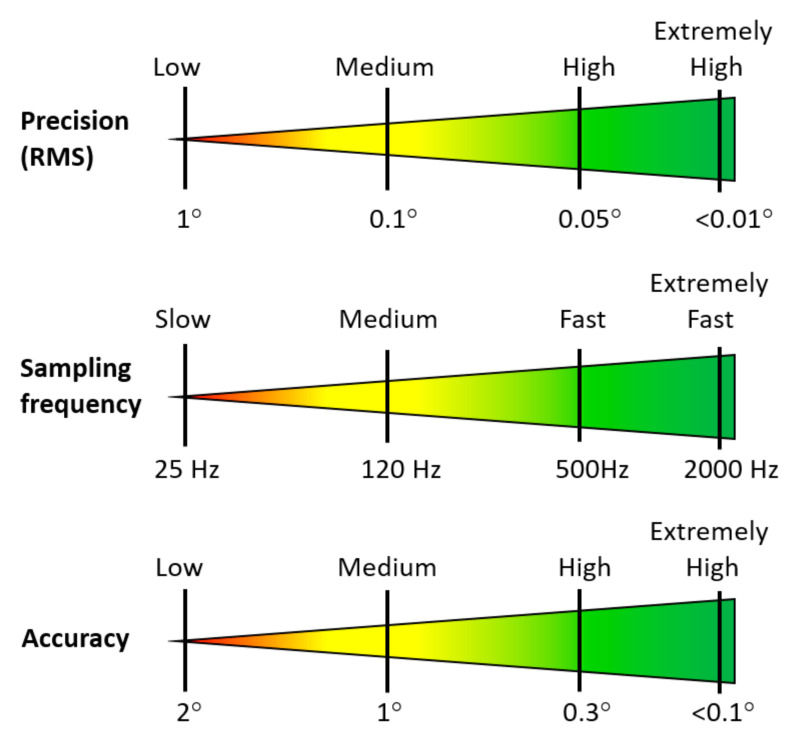
Performance specifications of current eye tracking systems.

The performance of eye tracking systems is primarily characterised by their sampling frequency, precision, accuracy, and the resolution of the system, as shown in Figure 8. Sampling frequency is the number of measurements taken by the system in one second (in Hz). Thus, a 100-hertz (Hz) eye tracker records a particular eye movement (or sample) 100 times per second [135]. Precision refers to the spread of the measured gaze points (in °). The accuracy refers to the difference between the measured and true eye position (in °) [140]. The saccade resolution is the ability of a system to detect saccade movement. For example, an eye tracker with 0.1° saccade resolution can detect movements as small as 0.1° [19]. The required performance of an eye tracking system relies on the type of movement that is detected; high-precision systems can accurately identify what a subject is looking at and where, while systems with a high sample frequency and high precision are more reliable in identifying the type of eye movement being observed [141]. For example, current high-tier systems offer sample frequency rates of 500 Hz to 2000 Hz, tracking accuracies below 0.3°, and precision better than 0.05° [108]. Mid-tier systems can provide sampling rates typically between 120 Hz and 200 Hz and have tracking accuracies of around 0.3° to 1° and precision of around 0.1° [142]. Low-end eye tracking systems typically have sampling frequencies of at least 30 Hz but reaching 120 Hz, tracking accuracies of 1° to 2°, and precision of more than 1° [141].

The performance specifications of commercial eye tracking systems are usually provided by their manufacturers/distributors. However, these values are obtained under ideal conditions and may be affected by the operating environment [19]. For example, several studies have reported large variations in the eye tracker’s accuracy compared to the values provided by the manufacturer [143,144,145]. Therefore, several studies have been performed to evaluate the performance of commercial eye tracking systems in different operating environments. For a detailed review about the methods for performance evaluation of eye tracking and technical characteristics of commercially available eye trackers, refer to the studies performed by Zhang et al. [146], Cognolato et al. [135], Lim et al. [142], and Stein et al. [147].

Data quality refers to the reliability, validity, and availability of the eye tracking data. In eye tracking, there is a large variety of factors that may influence the quality of data. One of these factors is the user’s neurology and psychology. For example, in dry eye syndrome, the use of glasses or contact lenses can lead to large data variation [148]. Environmental and light conditions can also affect the data collection processes. A very dry recording environment can change the rate at which an individual blinks, whereas changes in light conditions may lead to changes in pupil size [140]. Other factors that can significantly impact the precision and accuracy of the measures produced in an eye tracking experiment are the type of eye tracker used, the eye camera’s resolution and field of view, and the calibration procedure [149]. Therefore, to keep variation as small as possible, eye tracking studies try to carefully control eye tracking conditions and user positioning, frequently recalibrate the eye tracker, and exclude participants that do not track well [140]. While this is reasonable for research, it is not feasible in practice, limiting the use of eye tracking in everyday industry tasks. Thus, there is a great need to expand vision science beyond the controlled laboratory setting and into the natural world.

Equally important are the gaze estimation algorithm for data processing and the use of any filtering or pre-/post-processing algorithms. Currently, eye tracking algorithms have limitations and challenges to overcome. For example, the reliable classification of eye movements based on raw data is one of the challenges of processing algorithms. Moreover, there is a variety of different algorithms available today, which are frequently used without a systematic evaluation of their performance [150]. This makes it difficult to generalise research results when researchers use different hardware, algorithms, thresholds, and stimuli [150]. Another challenge is that most algorithms are bound to eye tracker settings and do not work even if the signal is not very noisy [151,152]. To overcome these limitations, several machine learning approaches have been developed to allow the algorithm to be retrained for any type of eye tracking system [140,153,154,155].

#### 3.5.4. Types of Eye Tracking Devices Used in Aviation, Maritime, and Construction

**Figure 9:**
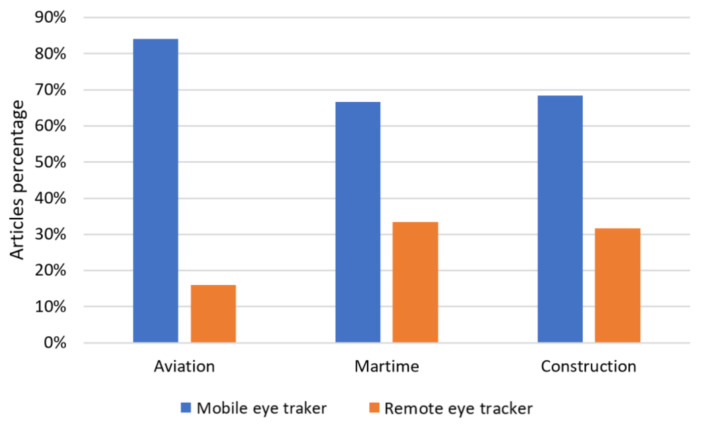
Preferred types of video-based eye tracking devices used in research for aviation, maritime, and construction applications.

According to our results, 100 percent of all the selected studies used some form of video-based eye tracking system. Based on Figure 9, mobile eye tracking devices are the preferred choice in aviation (84%), maritime (67%), and construction (68%) applications. We believe that the main reason is that mobile eye trackers are a flexible alternative that provides the user with freedom of movement, making them suitable for complex, dynamic, and real scenarios [8,16] such as those presented in these three industries. However, in simplified simulated environments in which the study environment is presented through a computer monitor, the preferred choice is a remote eye tracker [36]. We found that the majority of studies that used remote eye tracking systems in aviation (16%) and maritime (33%) scenarios were mainly in the context of air traffic control monitoring [7,18,38,48,56,65,69,76] and evaluation of computer systems and interfaces [36,37,80]. In the case of eye tracking research for construction applications, remote eye tracking systems were mainly used in studies that employed images presented in computer monitors to study visual search patterns [16,19,82,93] and for visual support systems [88]. Moreover, among all three industry sectors analysed, only one study in construction applications used tower-mounted eye tracking [86]. A summary of the advantages and disadvantages of video-based eye tracking system types for aviation, maritime and construction applications is provided in Table 5.

### 3.6. Application of Eye Tracking Technology in Aviation, Maritime, and Construction Scenarios

**Figure 10:**
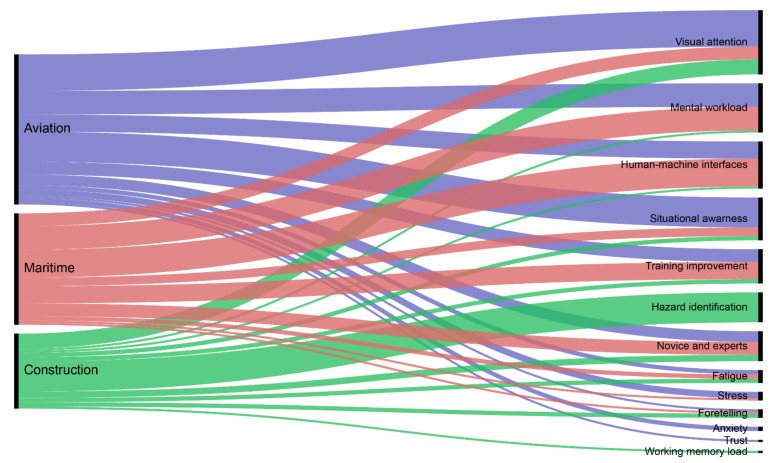
Different applications of eye tracking studies in the aviation, maritime, and construction industries (created with RAWGraphs [104]).

Video-based eye tracking technologies have been extensively used in various industry applications. In this study, we found that there are 13 main applications of eye tracking technologies for the aviation, maritime, and construction industries, as presented in Figure 10. In descending order, these applications were (1) visual attention, (2) MWL, (3) HMI, (4) SA, (5) training improvement, (6) hazard identification, (7) novice and expert comparison (8) fatigue, (9) stress, (10) foretelling, (11) anxiety, (12) trust, and (13) working memory load. The following 12 sections of the manuscript discuss the 13 main applications of eye tracking technologies; ‘stress and anxiety’ are combined under a single title.

#### 3.6.1. Visual Attention and Gaze Pattern

According to our results, visual attention is the most studied aspect in eye tracking research for maritime, construction, and aviation applications. Visual attention guides human perception, ensuring that an individual perceives and processes information selectively. Distraction occurs when attention shifts away from the original task [5]. Abundant evidence indicates that visual attention is essential for many cognitive tasks, such as attention distribution, hazard identification, decision-making, and SA [6,81,89]. Visual attention can also be used to compare tacit knowledge, such as scan patterns between novices and experts [32]. Moreover, working memory and its capacity are related to an individual’s ability to control their attention [86]. Based on our results, 56% of the eye tracking studies on visual attention and gaze pattern belong to the aviation industry, while the remaining 44% are almost equally distributed between the maritime (20%) and construction (24%) industries.

In aviation, Li et al. [74] studied air traffic controllers’ visual scan patterns to investigate the effectiveness of multiple remote tower operations. Their results showed that the visual scan patterns of air traffic controllers presented significant task-related variation while performing different tasks and interacting with various interfaces on the controller’s working position. According to Li et al. [74], air traffic controllers’ visual attention was influenced by the characteristics of the operating environment, how the information was presented, and the complexity of this information. For maritime applications, Li et al. [6] proposed a novel approach to assess trainees’ visual attention in a maritime operation simulator. For this purpose, expert knowledge was used to divide the task, identify critical operation, and define AOIs. An operation-dependent weighted attention map of the expert’s visual attention was then generated using their spatial and temporal perspectives. To test the effectiveness of the resulting attention map for training purposes, ten trainees were separated into two groups to assess their performance in a heavy lifting operation. The first group received detailed information about critical AOIs, the risks in operation, and the visual focus to ensure safety, whereas the second group only received information about the potential risks. According to their results, the second group had inferior visual focus, demonstrating the effectiveness of the debriefing provided to the first group based on the expert’s attention map. Li et al. [6] concluded from their results that their proposed method is valid to assist in training programs in maritime operations. 

In another study, Pinheiro et al. [16] studied workers’ gazing patterns during a hazard recognition task to understand the difference in visual patterns when a 2D sketch representation and real images of construction sites are used. Their results showed that when 2D sketch images are used, workers’ attention is considerably more dispersed, while, in the realistic image, the attention is more directed towards the AOI. The subjects spent 18% less time when observing 2D sketch images and the identification of hazards was faster. According to Pinheiro et al. [16], the use of 2D sketch images may be useful to introduce students and workers to the different types of hazards in a construction site. However, due to the complexity of a real construction site, 2D sketch images cannot fully prepare the students for a real scenario. Therefore, the use of mobile eye tracking systems during and after training sessions in real construction sites can help to analyse group patterns and develop new prevention measures. 

#### 3.6.2. Mental Workload

Mental workload refers to the mental effort required of an individual to perform a specific task [157]. MWL can be affected by cognitive, physiological, and emotional factors such as short-term memory, capacity, fatigue, and motivation. Therefore, mental workload is vital for the assessment of human performance. Excessively high or low MWL has a significant impact on an individual’s performance [158]. For example, in the aviation industry, it has been found that a pilot’s situation awareness is strongly correlated with MWL. Consequently, excessive MWL may result in poor situation awareness [159]. The advantages of using eye tracking instead of self-assessment methods to measure mental workload are the elimination of various human factors such as bias, likelihood of falsified results, likelihood of random responses, mistakes, and complaint attitudes [27]. Moreover, self-assessment methods cannot be used in real time, whereas eye tracking technologies offer the possibility of continuously monitoring an individual’s cognitive state without interfering with their performance in real-life situations [77]. 

Approximately 29% of all the studies measured mental workload for different purposes. Mental workload was the second most studied aspect for the maritime (48%) and aviation (48%) industries, whereas eye tracking studies in the construction industry showed little interest (4%) in studying this factor (Figure 10). The studies that applied eye tracking in the maritime and aviation sectors studied MWL in situations involving different emotional and physiological states and to understand the impact of HMIs on individuals’ MWL. For example, Yan et al. [27] studied the relationship between operators’ MWL and eye responses in the task of operating a marine engine interface. Moreover, they developed an artificial neural network (ANN) model to predict the operators’ MWL based on integrating eye response data. According to Yan et al.’s [27] results, eye response is sensitive to MWL when using the interface control. Furthermore, their ANN model presented high levels of prediction accuracy for the prediction of operators’ MWL based on eye response indices, with an R^2^ (determination coefficient) of 0.971, 0.912, and 0.918 for training, validation, and testing, respectively. For aviation applications, Martin et al. [56] studied MWL experienced by air traffic controllers during their work activity. Their results confirm previous studies’ results, showing that MWL increases when task requirements increase. Moreover, their results particularly highlight the crucial status of conflict in MWL and attention during air traffic control task execution.

By contrast, the construction industry had only one study that involved MWL and eye tracking devices. In this study, Li et al. [92] used eye tracking glasses to evaluate the impact of mental fatigue on construction equipment operators’ ability to detect hazards. For this purpose, they used the NASA Task Load Index in conjunction with eye tracking to measure operators’ perceived workload in six dimensions: mental demand, physical demand, effort, own performance, temporal demand, and frustration.

#### 3.6.3. Human–Machine Interfaces

With the rapid development of technology, sophisticated HMIs have evolved to facilitate complex operating procedures [160]. However, the design of HMIs can also negatively affect human cognitive performance. Therefore, to properly design an HMI with a human-centred design concept, it is first vital to understand the effects and interaction between humans and technology [43]. It has been found that the eye scanning pattern is one of the most robust methods for evaluating human cognitive processes when interacting with computers and machines [73]. According to our results, 27% of the studies used eye tracking to understand the impact of HMIs on individuals’ performance (Figure 10). However, it was found that only one study used eye tracking to study HMIs in construction applications. The lack of eye tracking studies applied to HMIs in construction can be explained by the lack of complex machine and computer interfaces in this industry. Construction equipment operation is relatively simple compared to the complex equipment in airplane cockpits and ship bridges. Most of the studies on HMIs were from the aviation and maritime industries, where eye tracking was mainly used to assess the impact of HMIs such as the cockpit (for airplanes) and the bridge (for ships) on individuals’ visual attention, gaze pattern, and MWL, as well as for training improvement [26,27,39,52]. For example, Li et al. [26] investigated pilots’ visual parameters to compare a traditional crew alerting system with a new, integrated system designed to assist pilots during urgent situations. They found that pilots’ visual parameters had significant differences while interacting with different types of displays showing numeric, symbolic, and textual messages. Li et al. [26] concluded that it is important to adopt a holistic approach for the design of flight decks to allow pilots to gain situation awareness rather than focusing on only one display. Hareide et al. [39] used eye tracking on board the Skjold-class Corvette ship, which is the world’s fastest littoral combat ship, and the exact replica of the Skjold-class corvette bridge to determine how to design better ship bridges and navigator interfaces. By analysing scanpaths and sequence charts, Hareide et al. [39] identified several bridge design factors that divert time and attention from the primary focus area of the navigator. Moreover, they found that the Route Monitor window in the graphical user interface is time-consuming, reducing the time spent looking outside. These findings provided valuable information for future work to facilitate the design of bridges and graphical user interfaces for combat ships.

#### 3.6.4. Situation Awareness

Situation awareness is one of the most dominant human factors to efficiently perform tasks in dynamic time-sensitive and safety-critical situations [89]. Numerous studies have confirmed that 88% of human-related causes of accidents in high-risk environments can be traced back to SA [5]. Situation awareness refers to an individual’s perception and comprehension of their surrounding environment within a defined volume of space–time and projecting their status in the near future [161]. An individual can only develop SA by paying attention to, perceiving, and processing the environment. However, SA can be affected by distractive factors and attentional demands that exceed an individual’s attentional resources [5,89]. Thus, SA is considered critical in scenarios that require sequences of multiple interdependent decisions in real time in a continuously changing environment such as hazard identification, error detection, and activity monitoring [38,162].

Our results indicated that SA is the fourth most studied aspect with eye tracking technology in aviation, maritime, and construction (see Figure 10). According to our results, SA in eye tracking research has mainly been studied in aviation, representing 39% of aviation studies. In the case of maritime and construction applications, eye tracking research has not focused much on SA, representing only 16% and 10% of studies, respectively. 

In construction, Hasanzadeh et al. used eye tracking in two different studies to measure workers’ real-time SA in a real scenario [4], as well as to understand the relationship between SA and visual attention under fall and tripping hazard conditions [89]. Using eye tracking, SA has been studied for a variety of maritime applications. For example, Sanfilippo [41] integrated a multi-layer and multi-sensor fusion framework with one of the world’s most advanced simulators of demanding offshore operations to improve SA as an integrated component of simulation training. Hareide et al. [44] used eye tracking to improve graphical user interfaces in the bridge displays of high-speed crafts; this was done to understand the impact of the user interface on navigators’ SA and workload [44]. 

In the aviation monitoring context, SA—also called mode awareness—is defined as ‘the ability of a supervisor to track and to anticipate the behaviour of automated systems’ [53]. Situation awareness is also closely related to an individual’s error recognition capacity, which depends on their ability to notice changes [81]. In aviation, 36% of the studies used SA mainly for monitoring and error recognition applications. For example, Moacdieh et al. [60] studied loss of SA to examine pilots’ automation monitoring strategies and performance, as well as to understand human–automation interaction. In another study, Björklund et al. [53] studied the effect of verbal callouts on SA, automation errors, and flight performance during simulated commercial flights.

#### 3.6.5. Training Improvement

Expert knowledge is usually externalised through courses, training programmes, and written material. However, tacit knowledge-sharing practices are rare and atypical in many industries [163]. Tacit knowledge such as know-how, know-what, and experience is mainly unconscious and unique to each individual [10]. As a result, extracting expert knowledge is challenging [164]. With the use of eye tracking technology, it is possible to extract and transfer expert knowledge to be used for the development of enhanced training programmes [20,22]. Several studies have demonstrated that experts garner similar visual patterns and problem-solving strategies over time, with little variance between individuals compared with novices [62,165]. This is important because it demonstrates that extracting and sharing expert knowledge such as visual problem-solving strategies is worthwhile for improving trainees’ performance in complex visual domains [62]. 

According to our findings, training improvement is the fifth most studied application of eye tracking in research in the maritime, construction, and aviation industries. Eye tracking for training improvement has mainly been used in the maritime industry and represents 32% of the maritime research articles collected, followed by the aviation and construction industries (16% and 10% of the articles for each industry, respectively). In the maritime industry, eye tracking technologies have been used for the evaluation of present and future human working environments. For example, Hareide et al. [40] used eye tracking to compare the visual focus of the navigator in onboard navigation and bridge simulators. Their results suggest that, despite the higher MWL required in simulator navigation training, simulators provide similar training outcomes to onboard navigation. In aviation, eye tracking was used by Robinski et al. [61] to identify differences in scanning techniques between helicopter pilots with different experience levels during landing training. They observed that, during take-off and landing, experienced pilots tended to use more helicopter instruments to retrieve information than inexperienced pilots, who assess conditions by looking through the window. Moreover, Robinski et al. [61] revealed that eye tracking feedback can enhance simulator training transfer and can be highly useful for real flights. 

Traditional safety training programmes usually do not properly determine why construction workers fail to identify safety hazards [166]. However, through eye tracking, experienced workers can assist in training novice workers to maximise hazard recognition performance and safety awareness. Taking this into consideration, Jeelani et al. [93] used eye tracking to provide personalised training to construction workers on visual search patterns and hazards. For this purpose, construction site images with visual attention maps were used to trigger self-reflection and improvement in novices. Their findings demonstrated that personalised training assisted eye tracking, improving construction workers’ hazard recognition performance by 35%.

#### 3.6.6. Hazard Identification 

According to our results, hazard identification is the sixth most studied aspect using eye tracking technologies (Figure 10). However, hazard identification has only been studied in construction scenarios and is the most studied aspect of eye tracking research in construction, accounting for 74% of the total studies. Since the construction sector is one of the most hazardous industries, hazard identification is fundamental to construction safety management [167]; as such, these results are not surprising. 

A hazard is defined as something that can cause detrimental effects. When hazards are unidentified, individuals are more likely to be exposed to unanticipated hazards, indulge in unsafe behaviour, and suffer disastrous injuries [93]. Because hazard identification is largely a visual search task [10,92], eye tracking technologies have been extensively used to understand individuals’ search patterns and visual attention. For example, a study found that workers who expend more time inspecting the worksite and devote higher levels of attention demonstrate superior hazard recognition [93]. Moreover, subjects with superior hazard recognition performance tend to focus less on noncritical distractors [91]. Additionally, Dzeng et al. [10] demonstrated that experienced workers spend more time searching for inconspicuous hazards than they do for obvious hazards. 

Hazard identification and risk perception are closely related factors. Risk can be defined as the probability that a hazard will occur. However, risk can be perceived differently between individuals [168] in two fundamental ways. Risk can be perceived using logic to anticipate a hazard’s effects and facilitate risk assessment and decision-making [169]. Conversely, in daily life, risk is mostly handled through instinctive and intuitive reactions deriving from an emotional perception of danger [169]. It has been found that risk perception is one of the factors that most affects individuals’ safety on construction sites. For example, Habibnezhad et al. [23] found that, contrary to construction workers with higher risk perception, workers with lower risk perception generally spend more time analysing a hazardous situation. A similar trend was also found by Hasanzadeh et al. [83], who studied the impact of safety knowledge on construction workers’ hazard detection. They found that experienced workers spend less time analysing hazards because they can identify them more quickly [23].

#### 3.6.7. Comparison between Novices and Experts 

Through experience, experts learn to organise simple thoughts in a more organised and conceptually richer way than novices. At the same time, with less experience, novices usually reason, solve, and perform tasks in a different manner to experts. Novices’ decisions are based on rigid rules, whereas experts rely on experience [76]. Because of their experience, experts have a deeper understanding that enables them to process more information and automatically apply it [76]. With eye tracking, it is easier to understand the differences between novices and experts in a variety of scenarios and situations that would be difficult to measure through other means. 

Based on our results (Figure 10), comparisons between novices and experts are the seventh most studied application of eye tracking research for the maritime, construction, and aviation industries. Approximately 17.5% of the selected studies used eye tracking to compare novices and experts. This application of eye tracking seems more attractive for maritime applications, followed by construction and aviation applications, representing 24%, 16%, and 14%, of the studies in each industry, respectively. For example, in a maritime application, eye tracking was used to investigate novice and expert maritime operators’ foci of attention during safety-critical maritime operations [34]. The results showed that novice ship operators tend to focus for shorter times and less frequently on the outside environment than expert operators. Moreover, maritime expert operators fixate more, which reflects their level of experience as they possess knowledge of what to look for and possible dangers to be aware of [34]. 

For construction industry applications, Hasanzadeh et al. [83] used eye tracking to identify the impact of safety knowledge, training, work experience, and injury exposure on construction workers’ attentional allocation and hazard detection. For comparison purposes, participants were divided into less experienced and experienced workers. Their results revealed that experienced workers tracked back more frequently to hazardous areas and spent less time exploring the hazardous areas. This behaviour shows that experienced workers have a better balance between processing and searching the scene than those with less experience [83]. In terms of safety training, their findings demonstrated no significant difference between the search strategies of workers with or without safety training. Workers with previous injury exposure behaved more cautiously and conservatively. Hasanzadeh et al.’s [83] results demonstrated that past injury exposure significantly impacts the cognitive processes of individuals and increases their risk awareness.

In aviation, air traffic controllers of different expertise levels were subjected to a series of radar screen tests to determine their visual problem-solving strategies. The results showed that individuals with higher levels of expertise more efficiently retrieved relevant information and used more efficient scanpaths than novices [62]. In another study, Skvarekova et al. [75] employed eye tracking to determine the differences in scanning techniques and attention distribution of experienced and inexperienced pilots during a precision instrument landing system approach and a non-precision non-directional beacon instrument approach. Their results revealed that experienced and inexperienced pilots’ scanning techniques differed considerably. Experienced pilots were able to scan each instrument faster and retrieve more information in less time, which gave them more time to detect errors. On the other hand, novice pilots made more mistakes and ignored some of the cockpit instruments.

#### 3.6.8. Fatigue 

According to Van Cutsem et al. [170], ‘Mental fatigue is a psychobiological state caused by prolonged periods of demanding cognitive activity’. Mental fatigue, stress, and strong emotions can hinder individuals’ SA when attentional demands exceed their attentional resources [5,89]. It is estimated that 20% of traffic accidents are caused by fatigue, making it one of the main contributors to transportation accidents [171]. Fatigue not only leads to the risk of falling asleep but also to decreased performance and attention, slower reaction times, memory lapses, and an increased risk of error [172,173,174]. There are several types of observable task- and sleep-related fatigue. Task-related fatigue can be active or passive. Active fatigue is caused by cognitively difficult tasks that require high mental effort. In contrast, passive fatigue is caused by the underload of cognitive processes, which is typical in monotonous work situations that require low mental effort [175]. Sleep-related fatigue is usually caused by the disruption of the individual’s circadian rhythm as well as environmental factors that reduce sleep quantity and quality [176]. It is therefore unsurprising that shift workers are especially susceptible to this type of fatigue [177]. However, according to Hopstaken et al. [178], increased motivation can counteract mental fatigue.

Fatigue detectors have recently received great attention in eye tracking research [48]. In this study, fatigue was identified as the eighth most studied application of eye tracking technology in the aviation, maritime, and construction industries, as presented in Figure 10. With eye tracking technologies, researchers have attempted to detect fatigue in different scenarios in real time. For example, Gupta et al. [176] developed a framework for monitoring submarine teams via online. The aim of the proposed framework was to determine the fatigue level of individuals 24/7 in maritime environments. From a total of 58 metrics, they identified three that can be used to identify individual fatigue state, team fatigue states, and social cohesion. According to Gupta et al. [176], the only feasible technology that they identified for measuring the different fatigue factors in a submariner environment was eye tracking. Nonetheless, the proposed framework for fatigue monitoring faces challenges regarding individual and team fatigue assessment, as well as in the design and consideration of the effect of a fatigue management system and countermeasures. 

In the construction industry, wearable eye tracking devices were applied by Li et al. [92] to evaluate the impact of mental fatigue on construction equipment operators’ ability to detect hazards. Their results demonstrated that operators’ ability to detect hazards and reaction time were significantly affected by mental fatigue. According to Li et al. [92], mental fatigue made it difficult for excavator operators to maintain adequate hazard monitoring performance for their surroundings and related details. In a more recent study, Li et al. [95] developed a novel methodology to identify and classify multi-level mental fatigue in construction equipment operators. The identification and classification of mental fatigue levels were achieved using a combination of the Toeplitz Inverse Covariance-Based Clustering method and the support vector machine (SVM) algorithm. Overall, these two studies demonstrate the feasibility and effectiveness of wearable eye tracking technology for construction equipment operators.

In the aviation industry, fatigue is seen as a safety threat for pilots and air traffic controllers, who often experience disruptions to their circadian rhythms due to night and shift work [57]. Nevertheless, the displays in smart technologies and the interfaces of modern flight instruments may also affect pilots’ circadian rhythms and cause eye fatigue and stress. To study the effect of artificial light from electronic displays on commercial pilots’ visual fatigue, Brezonakova et al. [78] used a wearable eye tracking device in a flight simulator with a modern glass cockpit. Their results verified that the backlight of digital displays in the cockpit can cause visual fatigue in pilots, as a result of constant eye adaptation and long exposure to the artificially illuminated environment [78]. Moreover, their results showed that pilots’ visual fatigue depends on the instrument display’s backlight intensity levels. This demonstrates the importance of setting the correct backlighting intensity during flights. In another study, Wang and Sun [57] proposed a framework for real-time fatigue measurement combining face recognition and eye tracking technologies. The proposed framework was based on the percentage closure of eyes (PERCLOS) value as the fatigue judgment index, which proved to be a suitable index for detecting fatigue in aviation practice.

#### 3.6.9. Stress and Anxiety 

Stress is a type of mental tension caused by uncontrollable situations [179]. Stress affects cognitive and emotional processes, thereby impeding individuals’ decision-making processes [180]. Individuals under stress tend to expend less time analysing information, relying instead on automatised intuitive reactions [181,182,183]. Individuals’ personal coping resources and situation demands determine their response to a stressful situation [184]. A situation is perceived as challenging by individuals when adequate resources to meet the situation are available. On the contrary, a situation is judged as a threat when the available resources are insufficient [185]. 

One of the negative emotions triggered by stress is anxiety [186]. Anxiety is composed of heightened autonomic nervous system activity and feelings of unease and tension [187]. Anxiety is also known to cause detrimental effects on psychomotor and attentional skills [188] by disrupting the balance between goal-directed and stimulus-driven attentional systems; thus, it causes a diversion of available processing resources from task-relevant to task-irrelevant stimuli [189].

Our findings suggested that stress and anxiety are the 9th and 11th most studied applications of eye tracking research, respectively. Stress has been studied with eye tracking in the maritime and aviation industries, whereas anxiety was only studied in aviation scenarios, as presented in Figure 10. For example, in aviation, eye tracking was used by Stankovic et al. [64] to study information sampling and decision-making under acute stress. Different participants were required to perform a modified version of the Matching Familiar Figures Test (MFFT). The MFFT is an established measure of cognitive impulsivity. Their results showed that, under stress, the participants made decisions before fully sampling all available information, thereby demonstrating more impulsive decision-making behaviour. According to Stankovic et al. [64], the implications of their study could help to improve the design of visual displays, information consoles, and warning systems in cockpits to reduce accidents in aviation emergencies. In another study, Vine et al. [68] examined the influence of stress on the performance of highly skilled commercial pilots. Their study involved an engine failure on take-off scenario performed in a high-fidelity flight simulator. Vine et al. performed a series of hierarchical regression analyses to examine the extent to which demand and resource evaluations predicted pilots’ performance. Their findings suggested that pilots who adopted a threat response to stress displayed increased randomness in scanning behaviour (entropy), higher search rates, and reduced ability to inhibit distraction, indicating increased disruptions to attentional control and poor performance. 

In a different study, Allsop and Gray [63] used eye tracking and a heart rate monitor to study the effects of anxiety on attention and gaze behaviour while interacting with complex, dynamic systems. Their study comprised an aircraft landing simulation in low-visibility conditions. Anxiety was multidimensionally manipulated by combining ego-threatening and evaluative instructions, monetary incentives, and immediate consequences for performance failures. The study found that anxious participants presented an increase in the randomness of scanning behaviour and in the percentage of dwelling time toward the outside world. According to Allsop and Gray [63], their results can help in implementing eye tracking technologies in aircraft warning systems to identify pilot anxiety during operational activity via visual scanning behaviour.

#### 3.6.10. Foretelling

The used of eye tracking for foretelling individuals’ choices and human performance was identified as the 10th main application of this technology and is mainly applied in dynamic decision-making (DDM) environments. Continuously evolving DDM environments such as hazard monitoring, air traffic control, and emergency responses require a series of multiple interdependent critical decisions to be made in real time [190]. To make good decisions in high-risk environments, intensive training in highly procedural scenarios is required [81]. According to Figure 10, eye tracking has been employed for foretelling purposes in maritime, aviation, and construction scenarios. 

Regarding applications in the maritime industry, Peysakhovich at al. [38] explored the applicability of oculometry to enable an abstract decision support system to foresee future decisions made by maritime operators. The participants had to monitor a radar screen to assess the level of threat posed by an aircraft by classifying it as hostile, uncertain, or nonhostile. Their results revealed that when participants identified a sign of threat, they revealed a higher task-evoked pupillary response and increased pupil diameter compared to nonhostile classifications. Peysakhovich at al. [38] concluded that fixation transitions and pupil dilation can help to predict the upcoming decision of the human operator by approximately half a second before the decision is made. 

In aviation, Hasse et al. [59] employed eye tracking to improve the selection of future monitoring aviation personnel. For this purpose, eye tracking data were used to assess participants’ monitoring and detection performance for automation failures. Hasse et al. [59] used defined AOIs and participants’ visual fixation to distinguish whether automation failures were promptly identified. Their results indicated that, during anticipation and detection phases, low performers demonstrated significantly lower fixation counts on all potentially relevant AOIs versus high performers. Furthermore, during detection phases, low performers demonstrated significantly shorter gaze durations on all potentially relevant AOIs during the anticipation phase than high performers. Hasse et al.’s [59] results demonstrated that eye tracking can be used to predict individuals’ failure detection performance to improve the selection of future aviation personnel.

Crane operation in construction sites is not an easy task. Crane operation operators face challenges such as load oscillation, control input lag, and depth perception in the radial direction [88]. To facilitate crane operation, in-vehicle visual support systems (IVVSs) equipped with external cameras can be used, informing the operator about the operation state [88]. Nonetheless, the impact of the new information provided to the crane operator is unknown. Taking this into consideration, Chew et al. [88] used eye tracking to improve the design of construction cranes’ visual support systems. For this purpose, they employed a crane simulator with various IVVS designs and developed a gaze analysis solution for dynamic AOIs. To estimate the subjective responses of users, the researchers used six different gaze metrics: sparseness of attention, maximal fixation duration, randomness, uniformity, the summation of these metrics, and the proportion of gaze on the IVVS. Finally, they demonstrated that, using the selected gaze metrics, it is possible to employ gaze behavioural analysis as an everyday IVVS design tool for nonexperts.

#### 3.6.11. Trust 

Trust and trustworthiness are considered difficult-to-measure characteristics in individuals [191]. Trust implicitly refers to one’s positive expectations towards others’ actions and the belief that there is a high probability that others will act or behave as expected [192,193]. Conversely, distrust is ‘negative and implies fear of the other’ [193]. The results of our study showed that trust is one of the least studied human aspects of eye tracking technologies, ranking 12th (see Figure 10). Only one study used eye tracking to study trust for applications in aviation. Gontar et al. [73] used eye tracking to understand the behaviour of commercial pilots confronted with a cyberattack. According to Gontar et al. [73], when an aircraft system reports technical problems, pilots usually anticipate the aircraft’s behaviour and the most adequate course of action. However, when an aircraft is under a cyberattack, pilots’ trust in the system may be compromised as attackers can use pilots’ standard procedures to manipulate their behaviour [73]. Their results showed that the presence of a cyberattack led to more incorrect decisions, increased pilots’ workload, and weakened trust in the system, without delayed responses to alarms. They concluded that a need exists for training programmes that increase pilots’ awareness of potential cyber threats, how an aircraft can be infected with malicious software, and how to resolve this type of situation. Moreover, Gontar et al. [73] indicated that cyberattack warning systems similar to virus scanners or firewalls must be developed and installed to inform pilots when parts of the system have been infected with malicious software.

#### 3.6.12. Working Memory Load

Working memory is understood as a system used by the brain to temporarily store and manipulate information for short periods of time [194]. The amount of information held at any given time within the working memory system is referred to as memory load [195]. According to Bouchacourt et al. [196], working memory “acts as a workspace on which information can be held, manipulated, and then used to guide behavior”. Working memory is vital for most cognitive processes and is closely related to attention [197]. Working memory abilities may be impaired due to mental and physical fatigue [198,199]. Moreover, multitasking is an important characteristic of working memory and can be inhibited when working memory capacity is reached [195]. For example, an impaired or high working memory load increases the likelihood of human error affecting an individual’s attentional allocation and hazard detection capacity [86]. 

Despite the high importance of working memory for most cognitive processes, this study identified that this is the least studied aspect in eye tracking research (13th place) in aviation, maritime, and construction applications. The only eye tracking study identified related to working memory load was by Hasanzadeh et al. [86], who investigated the impact of working memory load on the safety performance of construction workers. Several participants were subjected to a series of visual tests using images of a construction scenario to monitor their ability to detect hazards under low and high working memory loads. According to Hasanzadeh et al.’s [86] results, workers under high working memory load paid less attention to hazards compared with workers under low working memory load. Moreover, it was noted that workers’ ability to search for and identify hazards was inhibited when they experienced high working memory loads. Thus, high cognitive loads and working memory loads can influence individuals’ hazard detection skills. Several studies have suggested that attention and working memory load are the same entity [200,201,202]; this is important because a clear understanding of the relationship between attention and working memory load can help to design better strategies and training in order to reduce the rate of accidents in the construction industry.

### 3.7. Integrating Eye Tracking and Other Technologies for Evaluating Human Factors

Eye tracking technologies are mainly used to study different aspects of individuals’ cognitive processes. However, eye tracking technologies can also be integrated with different technologies to further study different physiological and emotional human aspects. This technology integration can be used to better understand individuals’ minds during different scenarios and behaviours as well as the relationship between the different human cognitive, emotional, and physiological factors. Taking all this into consideration, we identified which human aspects are the most studied in the aviation, maritime, and construction industries with eye tracking technologies, including the different technologies that were integrated for their study. 

**Figure 11:**
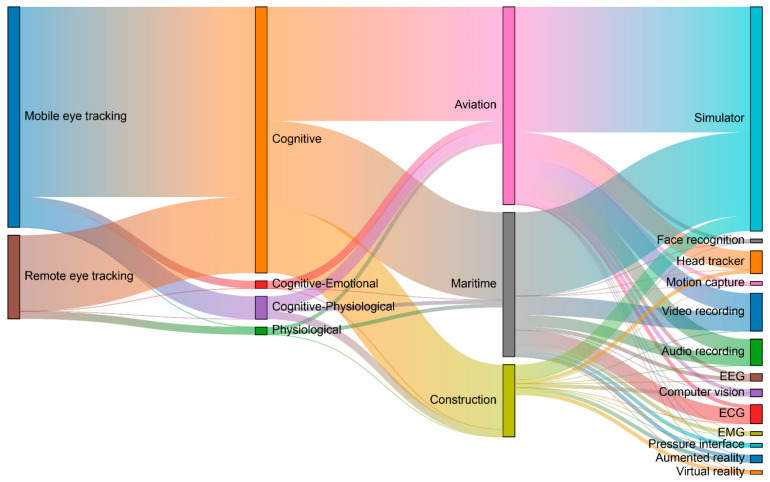
Different applications of mobile and remote eye tracking devices in relation to the study of human aspects, industry, and integration with various technologies.

From the two types of eye tracking devices available, we identified that 72.5% of all the reviewed eye tracking studies preferred to use mobile eye tracking devices, while the remaining 26.5% used remote eye tracking devices (Figure 11). The preference for using mobile eye tracking devices could be due to the complex scenarios encountered in the maritime, aviation, and construction industries. Moreover, to accurately simulate these complex scenarios, subjects need to move freely to allow the eye tracking device to capture the entire field of view.

Our results also showed that human cognition was the most studied aspect for aviation, maritime, and construction applications, representing a total of 87.5% of studies (Figure 11). The second most studied human aspect was a cognitive-physiological combination (7.5% of studies), followed by the cognitive-emotional and physiological aspects, each representing 2.5% of the studies. The study of the cognitive-physiological human aspects was mainly applied in the aviation industry, followed by the construction and maritime industries. However, the study of cognitive-emotional aspects was only applied in the aviation industry, whereas the physiological aspect was studied for applications in the maritime and aviation industries (Figure 11).

As mentioned, several studies have investigated the relationships among these aspects, such as cognitive–physiological and cognitive-emotional relationships. To capture these dynamics, these studies were required to integrate eye tracking with several technologies. We identified the use of the following 13 technologies: training simulators (74%), video recording (12.5%), audio recording (8.75%), head trackers (7.5%), electrocardiography (ECG; 6.25%), EEG (2.5%), computer vision (2.5%), augmented reality (AR; 2.5%), virtual reality (VR; 1.25%), pressure interface (1.25%), electromyography (EMG; 1.25%), motion capture (1.25%), and facial recognition (1.25%; see Figure 11).

According to our results, 74% of eye tracking studies preferred to use simulators to replicate real-world scenarios. Compared with real-world scenarios, simulators facilitate training, eliminate risks, and provide economic advantages. It was found that simulator training provides the same training outcomes as real-world scenarios [40]. However, to achieve this, simulators need to be as close to reality as possible [203]. Training personnel in simulators facilitate the overall understanding of the different operations to be performed [41] and the improvement of cognitive and psycho-motor skills, thereby increasing self-confidence. Moreover, simulation training can enhance communication and teamwork [5] and help to improve the design of graphical user interfaces [39]. 

#### 3.7.1. Simulators

The majority of eye tracking research applied in the aviation industry (92%) employed cockpit simulators. For maritime applications, 88% of eye tracking studies preferred the use of simulators to replicate ship bridges; in fact, international regulations for maritime training make simulators mandatory to use [49]. In the case of eye tracking studies for construction industry applications, only 21% used simulators to replicate construction sites or equipment operation cabins. Of the remaining eye tracking studies in construction, 26% used eye tracking in real scenarios, and 53% used pictures to represent construction sites. These findings are surprising as it has been demonstrated that two-dimensional images of scenes do not completely reflect the stimulus conditions of natural environments [87,89]. For example, it has been found that construction workers are not able to see all hazards in static images [23,91]. 

#### 3.7.2. Video and Audio Recording

Video recording is a technology used to assist eye tracking research by controlling the quality of the study or monitoring participants without causing disruptions [27,49]. Audio recording serves a similar purpose [27], but it can also be a beneficial source of information for debriefing [79]. Video and audio recording are simple but powerful technologies that assist eye tracking studies in further understanding and recreating the environment in which the studied subjects are tested. Moreover, these two technologies allow for monitoring and interacting with test subjects without physical interaction. 

#### 3.7.3. Head Tracking Systems

Head tracking systems can easily be integrated with mobile eye tracking devices. Head trackers are used in eye tracking studies to counteract some of the limitations of mobile eye tracking devices. For example, head trackers allow the orientation of the subject’s head to be tracked in space to calculate gaze as a 3D vector relative to the environment [53]. These data combined with eye tracking can be used to improve the accuracy of calculating the subject’s gaze areas, especially in environments where the subject moves freely. Moreover, knowing the position of the subject head facilitates the calibration, thus improving the accuracy of eye tracking process [19].

#### 3.7.4. Electroencephalography and Electrocardiography Technologies

It has been demonstrated that numerous mental states, such as vigilance, fatigue, alertness, stress, and performance, have physiological roots [57,204,205]. As a result, several mental states can be measured not only with eye tracking technologies but also with other techniques, such as EEG and ECG. Electroencephalography is a non-invasive method that uses electrodes placed on the subject’s scalp to sense brain cell activity. Brain cells communicate via electrical pulses. The number of neurons that discharge electrical pulses at the same time is workload- and task-dependent [77]; this allows EEGs to be used to study and monitor individuals’ mental and emotional states in real time during various activities [206,207]. Electroencephalography is so effective that it is considered one of the most powerful methods for monitoring task loads in real time. As a result, EEG has been used to evaluate the early onset of fatigue and drowsiness [24] as well as to validate the results of task load variations in eye tracking research [77]. Electrocardiography devices are used to visualise the electrical activity of the heart. Such devices allow the identification of different emotional and physiological states thanks to the relationship of the heart with the nervous system [208,209]. Since ECG can easily identify slight changes in normal ECG patterns, this technique is considered the most critical source of fatigue indicators [210].

#### 3.7.5. Body Pressure Mapping and EMG Systems

In human subjects, physical changes such as body posture and muscle activation can be used to identify certain mental states. For example, incorrect sitting posture can affect internal physiological conditions [211], leading to mental fatigue, impaired performance, and human error more generally [212]. The technologies used to measure body posture and muscle activation are body pressure mapping and EMG. Body pressure mapping, also known as body pressure imaging, is a technology that measures pressure distribution in the human body and support surfaces in real time [213]. Body pressure measurement systems are composed of a pressure mat fitted with an array of sensors that dynamically measure the interface pressure, indicating where pressure is concentrated [211]. This type of technology is usually used to help to assess comfort, design, and ergonomics in the automotive industry [214,215,216]. Electromyography is an electrodiagnostic medicine technique for evaluating and recording the electrical activity produced by skeletal muscles [217]. Electromyography measures the electric potential generated by contracting muscle cells when they are electrically or neurologically activated [218]. The information obtained using EMG can be used to inform eye tracking systems or to validate eye tracking data. For example, Fadda [24] used these two technologies in combination with eye tracking to understand fatigue evolution in quay crane operators.

#### 3.7.6. Computer Vision 

In eye tracking research, gaze behaviour is usually evaluated using qualitative methods such as heatmaps and gaze paths. However, the quantitative evaluation of gaze behaviour is one of the major challenges in eye tracking research [88] and is especially important in dynamic scenarios where the AOI is constantly moving or in computer displays with dynamically changing content. This challenge has been addressed by integrating computer vision tools with eye tracking software [58]. For example, the use of computer vision with eye tracking allows for the easy identification of exactly where a subject is looking in dynamic scenarios in real time. Chew at al. [88] used a computer vision tool to track the moving load of cranes to improve cranes’ IVVS. In another study, Weibel at al. [58] used computer vision to achieve eye gaze-to-object registration to collect, analyse, and visualise eye tracking data from pilots in commercial airline flight deck scenarios.

#### 3.7.7. Augmented Reality and Virtual Reality

Augmented reality has been extensively used for educational purposes [219]. Augmented reality technology adds virtual information to a real environment that is viewed through a device. Not only are users able to see and touch their natural surroundings, but they can also add virtual features such as images, videos, and sound [220]. Augmented reality has been applied in the construction industry to facilitate the comprehension of complex dynamic and spatial–temporal constraints, bring remote job sites indoors, and improve learning processes [90]. Augmented reality technologies have also been used in combination with eye tracking devices to improve HMIs by providing users with a more information-rich environment [37]. Virtual technology uses a head-mounted display helmet fitted with motion tracking sensors that allows users to engage in a fully immersive sensory experience in a designed space [221]. Virtual reality technology provides the user with an engaging experience by determining their spatial position in the visual environment, which is presented using 3D stereoscopic images and videos [222]. In recent years, VR has been rapidly recognised and implemented in engineering and medical education and training [222,223,224]. A combination of eye tracking with VR can be used to enhance the study of involved cognitive process and to improve the human–computer interaction [94]. For example, Ye et al. [94] used eye tracking and VR to demonstrate how integrating these technologies can enable the study of hazard identification in a realistic complex construction site by improving cognitive data collection and human–computer interaction.

#### 3.7.8. Motion Capture 

Motion capture is the process of recording human movement [225]. There are several types of motion capture systems, which are categorised into five groups based on their physical working principles. These principles include electromagnetic systems, image processing systems, optoelectronic systems, ultrasonic localisation systems, and inertia sensory unit systems [225]. In eye tracking research, there are situations where it is not possible to use traditional eye tracking systems to study gaze behaviour. These situations include when subjects use sunglasses or binoculars or when they need to move freely without using any equipment [226]. In such situations, non-invasive marker-less motion capture systems such as image processing systems can be used to determine an individual’s point of gaze [227]. Moreover, in relevant or special cases, extra information can be obtained by determining the position of the subject and their point of gaze [18,228].

#### 3.7.9. Face Recognition 

The human face is used as a biometric trait in various areas, such as human–computer interaction, health, education, security and law enforcement, entertainment, marketing, and finance [229]. Face recognition technologies can discreetly extract information from an individual’s face, such as emotions, mental states, race, identity, age, and gender [230,231]. Thanks to these capabilities, face recognition technologies have been used in conjunction with eye tracking devices to study consumer brand awareness [232] as well as fatigue detection in car drivers [233] and commercial pilots [57]. In this study, we identified only one study that combined face recognition and eye tracking techniques. Wang and Sun [57] proposed a framework that combines both technologies to measure fatigue in real time. According to Wang and Sun [57], the combination of these two technologies with the appropriate algorithms can result in a more effective tool to manage fatigue risks in aviation.

## 4. Discussion 

Human error is one of the main reasons for accidents in the aviation, maritime, and construction industries. While technological advancements assist in enhancing safety, the human operator still plays a key role in actively maintaining safety. It is the human operator’s responsibility to visually attend to and obtain information from available sources and to use it to maintain SA, identify hazards, and make appropriate decisions. This responsibility is particularly important as most accidents in these three industries can be linked to a lack of attention. Hence, eye trackers are a good way to measure an individual’s visual attention. The objective data provided by eye trackers make these a valuable technological tool for a variety of purposes, including research and training. The results of this research show that industries such as the aviation industry have been using eye tracking technology for longer and therefore have more pertinent articles than other industries, such as construction. Regardless, there has been an increase in the usage of eye tracking technology in the mentioned industries. The main countries using such technology can also be seen to be dependent on their location and industry resources. 

### 4.1. Gaps in Application

All three studied industries applied eye trackers to most of the topics identified in this study but at varying percentages. Topics such as fatigue were addressed within all three industries. However, other topics, such as MWL, were primarily studied by one or two industries. Finally, there were a few topics, such as working memory load, that were only studied within one industry. Such a trend reveals gaps in the literature. 

Topics such as HMIs and MWL have not been studied in depth in the construction industry. While the human operator might not have a complex workspace in the construction industry compared to the workspaces of the maritime or aviation industries, it is nonetheless important to conduct construction HMI studies utilising eye trackers. Such studies can aid in understanding how operators in the construction industry interact with their work interface and pay attention to vital information. Depending on the technologies used, the data could even reveal how workload is managed. Similar topics have been studied in the other two industries [26,27,39,52]. Such research might be particularly beneficial in construction as it is considered the most hazardous industry.

At the same time, topics such as hazard detection have been widely covered in the construction industry but not in the aviation or maritime industries [10,91,93]. Given that hazards are present in any high-risk industry, similar research might be beneficial to these industries. The dynamic operating environment of aircrafts and ships means that there might be even more hazards that the operator has to bear in mind. For example, moving vehicles (such as aircrafts and/or ships) depart one location and travel to another location; hence, they must monitor not only the hazards at the departure location but also hazards at the destination. Conducting studies on hazard detection in the aviation and maritime industries will shed light on how operators visually identify hazards in these dynamic environments. Such studies will show how operators focus on non-essential distractors and even obvious hazards. Similarly, there are applications of eye tracking research that have been studied less in one or two industries, revealing a gap in the literature. Eye trackers provide data about where a person is looking; hence, it is no surprise that visual attention is one of the most studied topics in all three industries.

### 4.2. Gaps in Human Aspects

The most studied human aspect in all three industries is cognition. However, it is possible to apply eye trackers to other topics as well, particularly in combination with other technologies, which will increase the literature on cognitive–physiological, cognitive–emotional, and physiological aspects.

Topics such as trust, working memory load, stress, anxiety, and so on could be investigated by integrating multiple technologies. However, some studies have only been conducted in one of the industries, revealing a gap in the literature regarding such studies on other industries. For example, anxiety is a relevant topic in most high-risk industries. Hence, a study similar to that of Allsop and Gray [63] could be conducted in the construction and maritime industries with the assistance of eye trackers to understand operator anxiety. Similarly, Hasanzadeh et al.’s [83] study could be replicated in aviation and maritime scenarios to understand the operator’s ability to pay attention to hazards under various working memory loads. This research is particularly relevant as the aviation and maritime industries do not have any eye tracking studies regarding hazard detection, as discussed in the previous paragraph. Moreover, applying multiple technologies will contribute to understanding the cognitive as well as other aspects of hazard detection.

### 4.3. Gaps in Technology Integration

In relation to technology, the technology consistently integrated with eye trackers for research in all three industries was simulators. Using simulators has obvious benefits, but the failure to integrate other technologies reveals a gap in the literature. Using additional technology could be done independently (one additional technology with eye trackers) or through integration with even more technologies (i.e., eye trackers and multiple other technologies). For example, a head tracker could be integrated with an eye tracker to obtain eye movement data along with head movement data. Additionally, EEG could be integrated, which could provide extra data on the operator’s mental state. These two examples are discussed further in the next paragraph.

To be able to make appropriate decisions, an operator must have a good scanpath. Studying an individual’s scanpath helps in understanding the overall process of gathering information from available sources to inform decision-making. As such, in a dynamic operating environment, integrating head trackers along with eye trackers can be beneficial because several workspaces in the three industries require the operator to move their head to scan relevant AOIs. Head trackers can further assist in mapping an individual’s gaze pattern and creating heatmaps. In a similar way, it could be valuable to integrate EEG technology with head trackers and eye trackers to provide an additional layer of objective data related to brain activity. Such data not only indicate where an individual is looking but also help with understanding the wider context of how the individual is using the obtained information to make decisions. This understanding is valuable because the acquisition and processing of information have close physiological links. We identified that EEG is not widely integrated with eye trackers, revealing a gap in the literature.

Likewise, pressure interfaces used to measure body pressure are not often used in eye tracking research. Integrating such technology with eye trackers might be beneficial to understanding other human aspects such as physiological conditions. Body pressure data are particularly relevant to the three industries in this paper as some roles might require the operator to spend a great deal of time sitting down. Depending on the role, operators might not have the flexibility to stand up and move around (e.g., a crane operator sitting in a small cabin for an extended period of time or a pilot sitting in a smaller aircraft during a flight lasting several hours).

The lack of literature using certain technologies in conjunction with eye trackers does not necessarily indicate a reluctance to use certain technologies. It could be that some technologies are new and have only been made available recently, such as AR and VR technologies. Augmented reality and VR could present a suitable alternative to simulators. As discussed, simulators are the most frequently integrated technology with eye trackers because of the advantages that they offer, such as low risk. Augmented reality and VR technology have several similar benefits and are also more portable, cheaper, easier to operate by one individual (i.e., the person wanting to use them), and require less maintenance. Moreover, AR/VR technology can even have inbuilt eye tracking technology, eliminating the need for additional hardware. While more studies are needed to determine whether VR can provide similar training outcomes to simulators [40,61], there is potential for using VR for learning and training purposes. Outside the aviation, maritime, and construction industries, these technologies are being used in other industries for learning purposes [222,223,224], and it might be possible to teach proper information gathering techniques to novices by experts [20,22,34,62,165] using the eye trackers integrated within AR/VR technologies. In this way, novices can learn to identify relevant information at the appropriate time and thus make correct decisions. Novice training could also become more efficient. Additionally, training could be offered to experienced operators as well through refresher trainings. Augmented reality and VR technologies can benefit such training syllabi, but the gap in AR/VR research means that further studies are needed to understand whether these benefits exist.

The application of non-invasive brain stimulation (NIBS) in conjunction with eye trackers is a technology integration that was not identified in this study. Non-invasive brain stimulation can induce persisting modifications of cortical excitability in humans [234,235], with beneficial effects on cognitive and physiological performance [236,237,238]. For example, Waters-Metenier et al. [239] showed in a double-blind experiment that transcranial direct current stimulation (tDCS, a type of NIBS) can be used to augment synergy learning, leading subsequently to faster and more synchronised execution of difficult muscular activation patterns when playing the piano. Their results demonstrated that NIBS could be used to facilitate and speed up the learning process of individuals in complex multitasking activities. In another study, Ciechanski et al. [240] used tDCS to enhance the motor skill learning of medicine students for surgical procedural training. According to their results, tDCS may enhance skill acquisition in a simulation-based environment. Taking into consideration the reported beneficial effects of NIBS, NIBS integrated with eye tracking can represent a suitable alternative to enhance individuals’ working memory and knowledge acquisition during training programs in high-risk industries. For example, this technology integration can facilitate the skill acquisition process of future pilots for complex operating procedures in aviation. In the construction industry, eye tracking and NIBS can be used to improve construction workers’ hazard recognition capability. Since modulation of neuroplasticity can be achieve with NIBS [241], this technology can be used to improve individuals’ working memory function [237] to shorten the learning curve in training programs.

### 4.4. Gaps in Types of Eye Trackers Used

As mentioned, simulators are the most used technology in all three industries for eye tracking research. There are many reasons for this, including regulatory requirements in the maritime industry [49]. The construction industry does not use simulators as much as the other two industries, but several real-world construction industry studies have used eye trackers; this reveals a gap in the literature regarding using eye trackers in real-world situations for the aviation and maritime industries. Mobile eye tracking devices and remote eye tracking devices have been used in studies of the three high-risk industries. There are obvious reasons for using a mobile device in an operating environment where the individual needs to be able to move their head freely. For example, the three high-risk industries have a broad view of the outside world that the human operator has to monitor from their workspace (e.g., the bridge of a ship) in addition to various screens. The number of displays, the type of information they present, and the frequency at which the operator monitors these displays vary considerably between the three industries and could also be dependent on the operator’s role. The gap in the literature regarding real-world situations, along with the low usage of remote eye tracker devices, indicates that more research is needed in this area. It might be possible to permanently install remote eye tracking devices using the screens in certain workplaces in order to collect data discreetly in real-world situations. Despite the limitations of such devices, such as the inability to collect eye movement data beyond the screens (e.g., when the operator is looking at the outside world), remote eye tracking devices could be a suitable option for real-world situations [121,138]. Such devices could provide real-time eye tracking data to operators, describing fixation, saccades, and blink rate, which could then be used to produce a collective heatmap [89]. Remote eye tracking devices also offer other benefits, such as collecting objective real-time data whilst the operator is completing a task [77], which could be used to identify anxiety, stress, or fatigue, as in Allsop and Gray’s study [63]. 

One additional benefit of permanently installing remote eye tracker devices in a workspace is the ability to collect longitudinal data, which could assist developers in monitoring eye tracking data over a long period of time to determine if enhancements to the HMI are needed. Data can be used to produce a collative heatmap and monitor gaze patterns over several years [53,115], revealing any limitations of the screen displays over the course of executing a given task. As discussed, understanding how humans interact with systems is vital to having a suitable HMI [43]. Remote eye trackers have the potential to provide real-time data that can assist in long-term system development.

## 5. Conclusions

In 1596, Du Laurens, a French anatomist and medical scientist, said that the eyes are the windows of the mind [242]. In high-risk industries, it is vital for humans to use their sight to obtain the right information at the right time, from the right sources. Hence, eye tracking technology is a valuable tool for collecting relevant objective data. While eye tracking technology has a long history, its uses are constantly evolving. 

This study systematically identified the demographic distribution and applications of eye tracking research in the aviation, maritime, and construction industries, as well as the different technologies that have been integrated to study human aspects of various high-risk environments. We also uncovered various gaps regarding the usage of additional technologies to support and validate eye tracking research for applications in the aviation, maritime, and construction industries. 

These gaps provide insight into some of the future eye tracking topics that could be explored in these three industries. Moreover, it may be beneficial for future research to explore the application of eye tracking in other high-risk industries, such as space exploration, mining, and oil and gas. Further research could help to reveal additional similarities and differences between different applications of eye tracking in the three industries.

However, eye tracking does not always translate smoothly into the real world. To achieve an adequate translation, several challenges need to be overcome in terms of data quality and algorithmic variability. Therefore, future work is necessary to create appropriate experimental and industry standards for eye tracking technologies.

Overall, this study highlighted that eye tracking can be used in relation to different human aspects for a variety of applications and industries. Eye tracking research has an exciting future, particularly in light of potential technology integrations such as the integration of NIBS, VR, and AR.

## Figures and Tables

**Table 1:** Search strategy, custom range 2000–2020.

Database	Records Identified	Total
Google Scholar	48,760	50,777
Science Direct	2017	
Duplicates	3617	47,160

**Table 2:** Classification of the selected classification of eye tracking studies.

Ref	Code	Year	Location	Maritime	Aviation	Construction	Cognitive	Emotional	Physiological	Tech Integration
[29]	S1	2007	Sweden	✔						
[30]	S2	2010	Germany	✔			✔			
[31]	S3	2012	Norway	✔			✔			✔
[32]	S4	2012	Sweden	✔						
[33]	S5	2013	Poland	✔			✔			
[34]	S6	2014	Norway	✔			✔			
[35]	S7	2014	UK	✔			✔			
[24]	S8	2015	Italy	✔			✔		✔	✔
[36]	S9	2015	Canada	✔			✔			
[37]	S10	2015	Singapore	✔			✔			✔
[38]	S11	2015	Canada	✔			✔			
[39]	S12	2016	Norway	✔						
[8]	S13	2016	Italy	✔			✔			
[40]	S14	2016	Norway	✔			✔			
[41]	S15	2017	Norway	✔			✔			✔
[42]	S16	2017	USA	✔			✔			
[43]	S17	2017	Norway	✔						
[44]	S18	2017	Norway	✔						
[45]	S19	2018	Australia	✔			✔			✔
[46]	S20	2018	Norway	✔			✔			✔
[47]	S21	2018	Sweden	✔						
[48]	S22	2019	Singapore	✔					✔	
[27]	S23	2019	China	✔			✔			✔
[6]	S24	2019	Norway	✔						✔
[49]	S25	2019	Turkey	✔			✔			
[50]	S26	2003	USA		✔					
[51]	S27	2004	USA		✔		✔			
[52]	S28	2005	USA		✔		✔			
[53]	S29	2006	Sweden		✔		✔			✔
[54]	S30	2007	USA		✔		✔			
[55]	S31	2009	Germany		✔		✔			
[56]	S32	2011	France		✔		✔			
[57]	S33	2011	China		✔				✔	✔
[58]	S34	2012	USA		✔					✔
[59]	S35	2012	Germany		✔		✔			
[60]	S36	2013	USA		✔		✔			
[61]	S37	2013	Germany		✔		✔			
[62]	S38	2014	The Netherlands		✔		✔			
[63]	S39	2014	UK		✔		✔	✔		✔
[64]	S40	2014	UK		✔		✔		✔	
[65]	S41	2014	Germany		✔		✔			
[66]	S42	2015	USA		✔		✔			
[67]	S43	2015	Germany		✔		✔			
[68]	S44	2015	UK		✔		✔		✔	
[69]	S45	2015	Germany		✔		✔			
[5]	S46	2016	Switzerland		✔		✔			
[70]	S47	2016	UK		✔		✔			
[71]	S48	2017	France		✔		✔			✔
[18]	S49	2017	Hungary		✔		✔			✔
[72]	S50	2018	USA		✔		✔			
[73]	S51	2018	Germany		✔		✔	✔		
[74]	s52	2018	UK		✔		✔			
[75]	s53	2019	Slovakia		✔		✔			
[76]	S54	2019	Germany		✔		✔			
[77]	S55	2019	Spain		✔		✔			✔
[78]	S56	2019	Slovakia		✔		✔		✔	
[26]	S57	2019	UK		✔		✔			
[79]	S58	2019	Switzerland		✔			✔		✔
[80]	S59	2020	Switzerland		✔					
[7]	S60	2020	China		✔					
[81]	S61	2020	France		✔					
[82]	S62	2015	USA			✔				
[19]	S63	2015	USA			✔				
[83]	S64	2017	USA			✔				
[10]	S65	2016	Taiwan			✔				
[16]	S66	2016	Brazil			✔				
[4]	S67	2016	USA			✔				
[23]	S68	2016	USA			✔	✔			
[84]	S69	2017	USA			✔	✔			
[85]	S70	2015	USA			✔	✔			
[86]	S71	2017	USA			✔	✔			
[87]	S72	2018	USA			✔				
[88]	S73	2018	Japan			✔				✔
[89]	S74	2018	USA			✔	✔			
[90]	S75	2018	China			✔				✔
[91]	S76	2019	China			✔	✔			
[92]	S77	2019	China			✔	✔		✔	
[93]	S78	2019	USA			✔	✔			
[94]	S79	2019	Germany			✔				✔
[95]	S80	2020	China			✔	✔		✔	

**Table 3:** Locations where eye tracking research was conducted on the aviation, maritime, and construction industries.

Continent	Location/Region	Number of Articles
South America	Brazil	1
North America	USA	20
	Canada	2
Europe	Germany	10
	Norway	9
	United Kingdom	7
	Sweden	4
	France	3
	Switzerland	3
	Italy	2
	Slovakia	2
	Hungary	1
	Poland	1
	Spain	1
	The Netherlands	1
Asia	China	7
	Singapore	2
	Japan	1
	Taiwan	1
Australia	Australia	1
Middle East	Turkey	1

**Table 4:** Summary of eye metric characteristics.

Eye Measure	Characteristics		
	Movement Rate	Latency	Relation to Individuals’ Functional State
Fixation	<15–100°/ms	180–300 ms	Attention, acquisition of information
Saccade	30–700°/s	20–200 ms	Attention and visual search
Change in pupil diameter	4–7 mm/s	140 ms	Cognitive workload, information processing, fatigue
Blink	12–15 per min	300 ms	Attention, stress, fatigue

**Table 5:** Advantages, disadvantages, and ideal applications of video-based eye tracking system types [122,156].

Eye Tracking Device Type	Ideal Application	Advantages	Disadvantages
Mobile	Aviation: Real-world applications and realistic cockpit simulators. Maritime: Real-world applications and realistic bridge simulators. Construction: Real-world applications such as construction site.	• Lightweight. • Can be fitted with a head tracker. • Provides freedom of movement, ideal for a real-world environment or realistic simulators. • Has cameras that records the scene image or environment.	• Sunlight may affect the quality of the data collection. • Gaze mapping is more challenging. • Gaze estimates are typically less accurate than those from remote systems. • Prone to movement, causing drifting. • Requires more recalibrations than remote systems.
Remote	Aviation: Ideal for simplified computer-based simulators. Maritime: Ideal for simplified computer-based simulators. Construction: Ideal for computer-based simulators of excavators or cranes.	• Ideal for on-screen studies on PCs, laptop monitors, and simulators. • Provides a good level of experimental control. • High accuracy and data quality.	• Sunlight may affect the quality of the data collection. • Experimental results cannot reflect the realistic and natural movements present in complex scenarios.
Remote with head-supporting towers	Aviation, maritime, and construction: when the accuracy and saccade resolution are the most important. For example, detection of micro saccades.	• Minimises artifacts caused by head movements. • Provide the greatest data quality and high level of experimental control. • Ideal when the accuracy and saccade resolution are the most important. • The saccade resolution is two to five times more than remote eye trackers. • Facilitates the calculation of the positions of stimuli on the monitor.	• Sunlight may affect the quality of the data collection. • Further constrains the subject of realistic and natural head movements.

## Data Availability

Data sharing not applicable. The new data created has already been presented in this study.
